# Thlaspi Bursa Pastoris in Hæmorrhage

**Published:** 1853-12

**Authors:** 


*Thlaspi bursa pastoris in Hæmorrhage.* By Dr. HANNON.—The cruciferæ arrange themselves, according to their therapeutical constituents, into two groups :—1st. Those which contain a sharp essential oil, and are, on that account, used as an irritant to the skin, such as mustard, etc. 2ndly. Into those which contain less essential oil, but, on the other hand, much astringent and bitter matter, and which are, therefore, useful in assisting digestion and improving the state of the blood. The number of the latter is very great. The whole coast of Northern Europe bears the cochlearia officinalis, a most useful antiscorbutic. The whole surface of Europe produces the nasturtium officinale, cardamine pratensis and amara, and lepidum sativum (garden cress.) On the coast of America and the Antilles is found kakile Americana,—another antiscorbutic of great value; the Laplanders, the Kamtschadalen, and the Greenlanders possess their nasturtium palustre and sylvestre.

All these plants possess an essential oil, containing sulphur, a bitter resin, tannic acid, salts of soda, potash, lime, and iron, with a nitrogenous substance, which, in decomposition, causes the evolution of ammonia.

The thlaspi bursa pastoris was used in the earliest periods, according to Dioscorides, as a remedy in hæmoptisis; in later times it was recommended by Simon Pauli, and recently it has been approved by Lejeune, Merst, and Delens, for the same disease, and by Dubois for hæmaturia and hæmoptisis. Rene Van Oye and Rademacher confirm its utility.

The author found it useful in many diseases, and especially in those hæmorrhages in which the fibrin of the blood is diminished. In certain subjects, the flow of blood constitutes the only disease; it occurs spontaneously, and without fever. The patients have, during the first attack, the aspect of good health, a fine rosy skin, and are well nourished; but upon closer inspection there is remarked a want of energy in the organic functions, labored and slow movements, and general apathy. By frequent recurrence of the hæmorrhages, there ensues pallor of the tissues, well pronounced anæmia; the nervous system seems over excited. Hence follow pains in the joints; the least pressure is followed by ecchymosis, in the form either of petechiæ or purpura; the least wound bleeds obstinately, and requires the repeated application of astringents; coagulation of the blood ensues with difficulty. In women the catamenia are frequent, profuse and exhausting. The blood appears serous and pale,—the clot is soft, small, and never buffed. The skin perspires easily and abundantly; every breath of air calls forth neuralgia. The urine is pale and copious; the evacuation from the rectum, fluid; and hydropic effusions, such as œdema of the foot, occur readily. The thlaspi bursa pastoris, administered for a considerable time, works in these cases a change in the constitution of the blood, especially when aided by the influence of good air and properly regulated diet. The best formulæ are the succus thlaspi, prepared cold, and of bitter taste, three to six drams a day. The extractum thlaspi, the expressed juice, reduced by evaporation to the consistence of an extract, one to two drams a day.—*Med. Times, from Presse Med.*, 1853.

